# iTRAQ-Based Quantitative Proteomic Analysis Reveals Changes in Metabolite Biosynthesis in *Monascus purpureus* in Response to a Low-Frequency Magnetic Field

**DOI:** 10.3390/toxins10110440

**Published:** 2018-10-29

**Authors:** Jialan Zhang, Yingbao Liu, Li Li, Mengxiang Gao

**Affiliations:** 1 College of Animal Science, Yangtze University, Jingzhou 434025, China; zhangjl@yangtzeu.edu.cn; 2 College of Life Science, Yangtze University, Jingzhou 434025, China; liuyingbao@yangtzeu.edu.cn (Y.L.); lily2012@yangtzeu.edu.cn (L.L.)

**Keywords:** LF-MF, *Monascus purpureus*, protein expression, citrinin, pigment, monacolin K

## Abstract

Background: Low-frequency magnetic fields (LF-MFs) dampen the citrinin output by *Monascus purpureus* in fermentations. The influence of LF-MFs on biosynthesis by *M. purpureus* was evaluated at the protein level. Methods: Cultures were treated with a 1.6-mT MF from day 0 to day 2 of incubation, and secondary metabolite production was evaluated on the day 12 of incubation. All proteins were extracted from *M. purpureus* mycelia and subjected to isobaric tags for relative and absolute quantification (iTRAQ) labeling and subsequent liquid chromatography/mass spectrometry (LC-MS/MS) analysis on day 6 of fermentation. Results: There was no difference in biomass between the treated samples and the control. Citrinin production was 46.7% lower, and the yields of monacolin K and yellow, orange, and red pigment were 29.3%, 31.3%, 41.7%, and 40.3% higher, respectively, in the exposed samples compared to the control. Protein expression in *M. purpureus* under LF-MF treatment was quantified using iTRAQ technology. Of 2031 detected proteins, 205 were differentially expressed. The differentially-expressed proteins were subjected to Gene Ontology (GO) functional annotation and statistical analysis, which revealed that they mainly refer to biological metabolism, translation, antioxidant, transport and defense pathways. Among all the tagged proteins, emphasis was placed on the analysis of those involved in the synthesis of citrinin, pigment and monacolin K was emphasized. Conclusions: LF-MFs affected *Monascus* secondary metabolism at the protein level, and aggregate data for all the protein profiles in LF-MF-treated *Monascus* was obtained.

## 1. Introduction

*Monascus* is a production strain for traditional fermented products in China and Southeast Asia. During the growth process, *Monascus* can produce numerous secondary metabolites including natural edible pigments, monacolin K (a hypoglycemic active substance also known as lovastatin), γ-aminobutyric acid (GABA, a component that lowers blood pressure), dimerumic acid (a natural antioxidant), acetylcholine, ergosterol, and citrinin (a nephrotoxin) [1,2,3,4]. Among these metabolites, monacolin K, the main component of functional red yeast rice, has been successfully developed as a lipid-lowering drug [5]. Other high-content functional ingredients of red yeast rice have been used as raw materials in the development of a variety of health foods [6]. In 1995, French scholars found that some *Monascus* strains can secrete citrinin, which contaminates red yeast products [7]. Through nearly 20 years of continuous effort, researchers have learned to effectively control the citrinin content of bacterial and production processes.

The optimization of fermentation conditions and breeding methods are currently used to control citrinin content by improving the yield of *Monascus* pigment, monacolin K, and other beneficial metabolites. The fermentation conditions that have been studied include the carbon source, nitrogen source, fatty acid content, temperature, ventilation conditions, pH, and other culture conditions [8,9,10]. The yield of beneficial metabolites can also be improved through the use of mutant strains. Methods for generating mutant strains include UV mutagenesis [8,11], ^60^Co irradiation, fast neutron irradiation, NTG (*N*-methyl-*N*′-nitro-*N*-nitrosoguanidine), EMS (ethylmethanesulfonate), and other chemical means [11,12]. Genetic engineering technology has been used to build mutant strains. This technology has been developed to regulate pigment [13,14], monacolin K [15,16,17], and citrinin content [18,19,20], as well as biosynthetic pathways. However, results for *Monascus* as a special species remain very limited, and there is still much room to further develop this organism.

A low-frequency magnetic field (LF-MF, <300 Hz) is a form of non-ionizing radiation. The optimum influence of MFs on microbes can be determined by artificially controlling the MF strength. This method is selective with a short cycle and great potential. LF-MFs can change the metabolic pathways of *Lactococcus lactis* [21], *Saccharomyces cerevisiae* [22], *Rhodotorula glutinis* [23], *Aspergillus niger* [24], *Monascus purpureus* [25], and α-hemolytic *Streptococcus* [26].

In preliminary experiments, LF-MFs dampened the citrinin output by *M. purpureus* in liquid-state flask fermentations [25,27]. In this study, the hyphae of the treatment and control groups were collected for isobaric tagging for relative and absolute quantitation (iTRAQ)-based proteomic analysis. Additionally, the down- and upregulation of a high percentage of the proteins revealed that LF-MFs can directly or indirectly alter biosynthesis. This is the first proteomic-level study to explore the molecular mechanisms by which LF-MFs affect *Monascus* secondary metabolism.

## 2. Results

### 2.1. LF-FM Treatment Influences Biomass, Citrinin, Pigment and Monacolin K Yield

**Figure 1:**
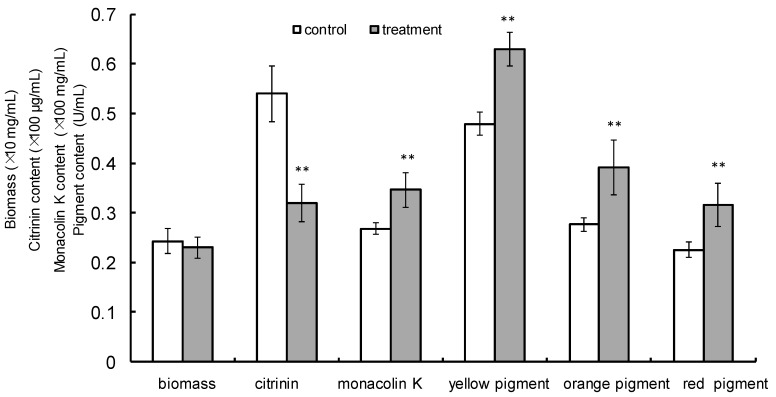
Biomass, citrinin, monacolin K, and pigment production by *M. purpureus*. The cultures were treated with a LF-MF (1.6 mT) for the first two days of incubation time and then continued to culture until day 12 at 30 °C and 150 rpm. The data represent the mean ± SD of six replicates. **, *p* < 0.01.

*M. purpureus* was incubated in a liquid fermentation culture medium and treated with a 1.6-mT MF for the first two days of incubation. After MF treatment, the cultures were incubated at 30 °C until day 12 of fermentation. Biomass, citrinin, monacolin K, and yellow, orange, and red pigment production were each evaluated. The biomass did not differ between the treated group and the control (*p* > 0.05). The citrinin production was 46.7% lower (*p* < 0.01) than that of the control, but the production of monacolin K and yellow, orange, and red pigments was 29.3% (*p* < 0.05), 31.3%, 41.7%, and 40.3% (*p* < 0.01) greater than that of the control, respectively (Figure 1).

### 2.2. Identification of Proteins Differentially Expressed in Response to LF-MFs

**Figure 2:**
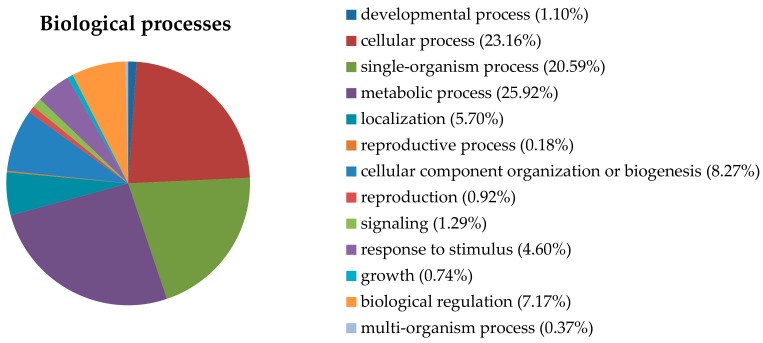

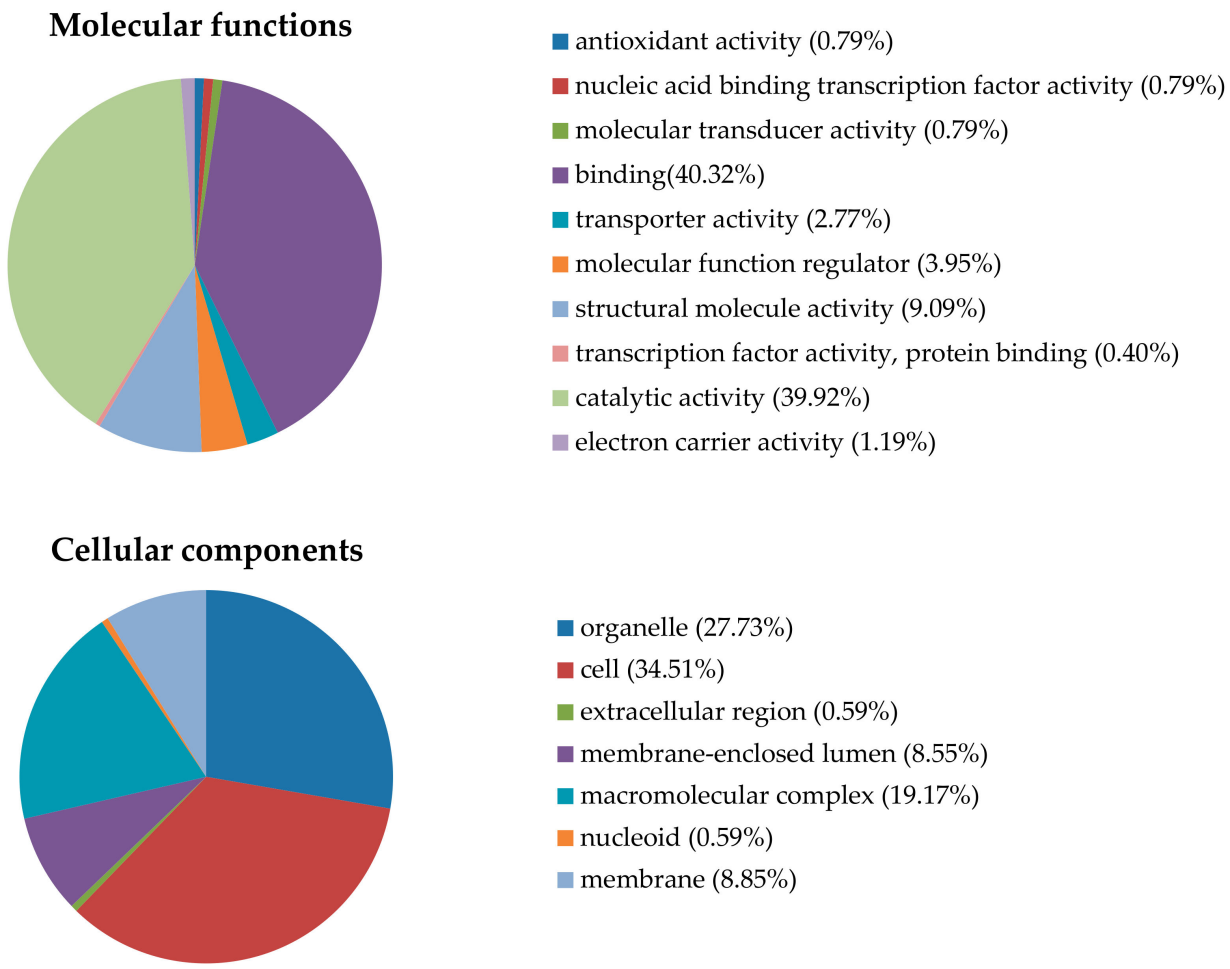
Subcellular localization and function of the *M. purpureus* proteins that were identified by iTRAQ proteomics analysis. The detected proteins were categorized into three types: Biological processes, molecular functions, and cellular components.

All proteins were extracted from *M. purpureus* mycelia (LF-MF treated (T) and untreated control (CK); three replicates in each treatment) and subjected to iTRAQ labeling and subsequent LC-MS/MS analysis. A total of 2031 proteins were identified in these samples, and these proteins refer to a large variety of biological processes, including metabolic processes (25.92%), cellular processes (23.16%), single-organism processes (20.59%), cellular component organization or biogenesis (8.27%), biological regulation (7.17%), responses to stimuli (4.60%), biological localization (5.70%), and signaling (1.29%) (Figure 2).

**Figure 3:**
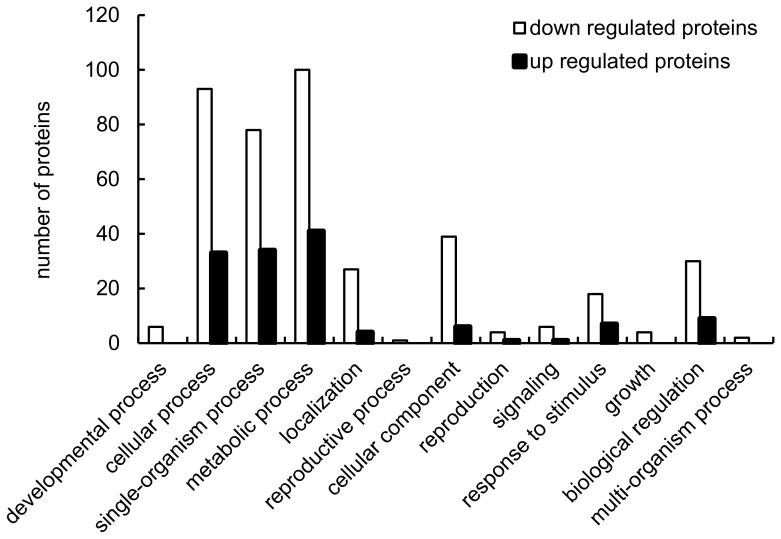
Functional classification of the differentially expressed proteins in response to LF-MFs based on their GO terms. The numbers of the proteins that were up- and downregulated in each category are shown.

We found that the protein expression levels of 61 and 144 proteins increased and decreased, respectively, when treated with LF-MF (Table S1 in Supplementary Materials). The GO analysis showed that many proteins that refer to the “metabolism process” were up- or downregulated, and several proteins involved in the “cellular process,” “single-organism process”, or “biological regulation” were also differentially regulated (Figure 3). Thus, LF-MF treatment strongly reshaped the *M. purpureus* proteome by affecting the physiology of *Monascus*, involving the metabolism, cellular processes, single-organism processes, and biological regulation. The top 10 up- or downregulated proteins included an enolase, a kynureninase or ribosomal protein, and salicylate hydroxylase, and these proteins are known to be important in metabolism.

### 2.3. Analysis of the Protein Families Associated with Citrinin, Pigment, and Monacolin K Biosynthesis

The proteins and genes involved in citrinin biosynthesis were all significantly downregulated (Table 1). Treatment with LF-MFs significantly reduced the production of citrinin biosynthetic genes at the translational level and resulted in a decline in citrinin production. The induction of proteins related to citrinin production (Aspzol_71435, Monrupul_468568, and Q4wyl4) was greatly reduced (0.629-, 0.774-, and 0.791-fold decrease, respectively) when the cultures were treated with the LF-MF. The level of biotin synthetase (Evm_model_C5_699) was the lowest of the proteins (0.404-fold decrease), suggesting that LF-MFs also influence citrinin production by affecting proteins related to citrinin-related metabolic pathways.

Conversely, the levels of the proteins and genes related to pigment and monacolin K biosynthesis were all significantly upregulated (Table 1). The levels of proteins directly related to pigment production (Mycgr3_71228, Monrupul_297557, and Aspwe1_68221) were all increased by LF-MF treatment relative to the control (2.032-, 1.615-, and 1.258-fold increase, respectively). Monacolin K biosynthesis-related proteins (Q5bcz6, Mycgr3_98821, and A0a0a2jfe4) also increased with LF-MF treatment (1.267-, 1.235-, and 1.208-fold increase, respectively). These results suggest that LF-MFs can alter *M. purpureus* metabolism by weakening the citrinin content and improving the yield of pigments and monacolin K without influencing growth.

## 3. Discussion

In total, there were 205 differentially expressed proteins, with 61 upregulated and 144 downregulated proteins, when the cultures were treated with LF-MF. These proteins are involved in biological metabolism, translation, antioxidation, transport, and defense pathways. The expression levels of the citrinin biosynthesis proteins CtnI (acyl-CoA synthetase), CtnH (short-chain dehydrogenase), and CtnR1 (WD repeat protein) were significantly reduced, indicating that the application of the LF-MF resulted in a significant reduction of the production of citrinin biosynthesis gene products at the level of translation (Table 1), which then led to a decline in citrinin production [15,18,19].

While genes and proteins related to citrinin biosynthesis were downregulated, the biosynthetic genes encoding proteins that produce pigments and monacolin K were upregulated (Table 1). These proteins were encoded by pigment biosynthesis genes (polyketide enoyl reductase (ER), esterase, fatty acid elongase, thioesterase, and P450 monooxygenase) [13,28] and genes involved in monacolin K biosynthesis (ketoreductase, *mokC* (P450 monooxygenase), and *mokH* (Zn(II)2Cys6 transcription factor)) [15,16].

In addition, the levels of mitogen-activated protein (MAP) kinase targets, of which several are proteins involved in citrinin metabolism, were drastically reduced. The MAP kinase pathway is a G protein signaling pathway [29]. G protein signal transduction pathways are involved in the control of the secondary metabolism of fungi [30,31]. G proteins have an impact on pigment and citrinin biosynthesis. When the G protein subunit gene is knocked out, pigment and citrinin production significantly increases. Overexpression of the G protein subunit can significantly decrease citrinin production, but has no effect on pigment production [31,32]. G proteins may negatively regulate pigment and citrinin production. G proteins partially regulate *Monascus* pigment and citrinin production via the cAMP-PKA pathway [32,33]. Regulators of G protein signaling (RGS) are GTPase-activating proteins (GAPs). The reactive oxygen species (ROS)-regulating protein MrflbA upregulates pigment and citrinin production by inhibiting G protein subunit activity [34]. The synthetic ability of the strain is significantly decreased when genes encoding proteins involved in either pigment or citrinin production are deleted. This result suggests that the G protein signal transduction pathway positively regulates pigment and citrinin production. The present study found that MAP kinase was stimulated by the LF-MF resulting in a downregulation of the G protein signal transduction pathway and leading to a significant decrease in citrinin production. The G protein positively affected citrinin biosynthesis, confirming previously published results. However, G proteins can also positively regulate citrinin production through the MAPK signaling pathway, proving that G proteins can partially regulate pigment and citrinin production by way of cAMP-PKA.

The expression levels of protein-related antioxidants (catalase and 1-Cys protein) were also significantly reduced. Thus, LF-MFs may perturb the redox balance of *Monascus*. LF-MFs can cause oxidative stress and increase ROS levels (including those of H_2_O_2_) [35]. It can therefore be inferred that these changes may also have potential links to the activity of antioxidant proteins and the ability to synthesize citrinin. Specifically, active oxygen will not be removed, leading to a decrease in citrinin synthesis because of the low expression levels of these antioxidant proteins.

Cytochrome c was also downregulated. Presumably, the loss of cytochrome c, which is the cytochrome c peroxidase substrate, may deactivate this peroxidase, leading to a decrease in citrinin synthesis. There is an 85% similarity between a T-DNA flanking fragment and the cytochrome c peroxidase gene in the *Monascus* strain mutant Mr-5 [36]. Citrinin production by this mutant is also dramatically smaller than that of wild type, indicating that cytochrome c peroxidase can improve citrinin production.

Among the downregulated proteins, biotin synthetase was the most downregulated. Biotin, also known as vitamin H or coenzyme R, is necessary for the synthesis of vitamin C and a necessary component in the homergy of fats and proteins. Biotin thus affects fatty acid production. Medium-chain fatty acids can enhance pigment and inhibit citrinin yield by *Monascus ruber* [37]. Therefore, pigment synthesis can presumably be improved via biotin by increasing the activity or amount of acetyl-CoA carboxylase. Acetyl-CoA carboxylase could then catalyze esterification polymerization to increase the amount of the common precursor (malonyl-CoA) in the pigment and citrinin biosynthesis. A decrease in the expression level of biosynthetic enzymes may decrease the activity or amount of acetyl-CoA carboxylase and thereby reduce citrinin production.

The LF-MF increased pigment and monacolin K production as well as decreasing citrinin production, possibly because citrinin, pigments, and monacolin K share the same precursors: acetyl- CoA and malonyl-CoA. After these compounds are produced from two ketone condensations, different synthetic branches are reached: specifically, one branch results in the formation of pigment and citrinin [38,39] and another that results in monacolin K [40]. Thus, once the route synthesizing citrinin was blocked, more precursors were channeled to the yield of pigments and monacolin K.

In addition, the expression of two pyruvate decarboxylases was upregulated by the LF-MF exposure, while the expression of acetyl-coenzyme A synthetase and biotin synthase was downregulated, leading to less acetyl coenzyme A and, in turn, lower amounts of the pigment and citrinin precursors acetyl-CoA and malonyl-CoA. This scarcity of precursors inhibited the synthesis of citrinin, pigments, and monacolin K. To synthesize these pigments, the precursor acetyl-CoA must be formed by related enzymes. Pyruvate carboxylase has three functions in eukaryotes: acetyl-CoA carboxylase, biotin carboxylase, and carboxyl transferase [41]. The LF-MF thus stimulated pyruvate decarboxylase to form acetyl-CoA followed by polymerization to malonyl-CoA, leading to a partition of polyketide metabolic flux from citrinin synthesis to pigment and monacolin K synthesis.

Many other proteins were differentially expressed as a result of the LF-MF treatment except the proteins discussed above; these proteins may be directly or indirectly related to citrinin, pigment, and monacolin K synthesis and should be identified for further study.

## 4. Materials and Methods

### 4.1. Inoculum Preparation and LF-MF Treatments

The *M. purpureus* stain SKY219 used in this study was obtained from the collection of the College of Life Science, Yangtze University (Jingzhou, China). The strain was cultivated in Czapek yeast extract agar (CYA) at 30 °C for 10 days. Then, the spore suspension was inoculated in potato dextrose broth (PDB) in proportion to 1:24, and cultivated at 30 °C and 180 rpm for 36 h. The concentration of the suspension was adjusted to approximately 10^5^ spores per milliliter. The inoculum was incubated for 24 h at 30 °C until day 6 of fermentation and was exposed to a MF intensity (the MF apparatus was described in detail by Zhang et al. (2015) [25]) of 1.6 mT for the first two days of incubation. A group without MF exposure was used as the control (the difference in metabolites between the exposure and control groups was obvious). All cultures were cultivated at 30 °C until day 12 of fermentation.

### 4.2. Determination of Biomass and Secondary Metabolites

The biomass was reported as the mycelial dry weight per unit volume of culture medium. The citrinin and monacolin K concentrations were determined by using high-performance liquid chromatography (HPLC). Yellow, orange, and red pigment concentrations were determined by measuring their absorbances on a spectrophotometer (UV1601PC, Shimadzu, Japan) at 410, 465, and 500 nm, respectively. The methods were adopted from Wan et al. (2017) [27].

### 4.3. M. purpureus Collection and Protein Extraction

The mycelia treated by LF-MF and the control mycelia were collected on day 6 of fermentation. The cells were frozen at once in liquid nitrogen and were ground into powder in a mortar with liquid nitrogen. The powder was homogenized with SDT buffer (4% sodium dodecyl sulfate (SDS), 1 mM dithiothreitol (DTT), and 150 mM Tris-HCl, pH 8.0), and heated for 5 min in a boiling water bath. Then, it was ultrasonicated 10 times (80 W, 10 s, ultrasonication with 15 s pause per time) and heated again for 5 min in a boiling water bath. It was then centrifuged for 45 min at 25 °C and 14,000× *g* to obtain the supernatant. The protein contents were determined by the bicinchoninic acid (BCA) method.

### 4.4. Protein Quantification and Digestion

For each sample, 200 μg of protein was mixed with 200 μL of UA buffer (150 mM Tris-HCl and 8 M urea, pH 8.0) and centrifuged at 14,000× *g* for 15 min to get precipitation. Next, the precipitation was mixed with 200 μL UA buffer and centrifuged again. Then, 100 μL of IAA (50 mM iodoacetamide in UA buffer) were added to the deposits and oscillated at 600 rpm for 1 min to block reduced cysteine residues, and then incubated for 30 min in the dark. The filters were washed twice with 100 μL of UA buffer and twice with 100 μL of DS buffer (50 mM triethylammonium bicarbonate, pH 8.5). The protein suspensions were digested with 40 μL of trypsin buffer (2 μg of trypsin in 40 μL of DS buffer) for 16–18 h at 37 °C, and were filtrated to collect the peptides. The peptide concentration was estimated using the UV light spectral density at 280 nm.

### 4.5. iTRAQ Labeling and Fractionation

Samples were treated using an iTRAQ reagent-8PLEX Multiplex Kit (AB SCIEX, Foster City, CA, USA) based on the manufacturer’s directions. Equal amounts of all treated samples were mixed and then fractionated using an HPLC system (Easy nLC1000, Thermo Finnigan, San Jose, CA, USA) equipped with a C18 column (5 μm, 200 A, 4.6 × 100 mm, Poly LC, Thermo Fisher Scientific, Waltham, MA, USA). Fifteen fractions were obtained.

### 4.6. LC-MS/MS Analysis

The LC-MS/MS analysis method used in this study was described by Xie et al. [42]. An AB SCIEX nanoLC-MS/MS system (Q-Exactive, Thermo Finnigan, San Jose, CA, USA) was used in the analysis. Samples were chromatographed using a 60-min gradient from 0% to 100% of mobile phase B in mobile phase A (mobile phase A: 0.1% (*v*/*v*) formic acid; mobile phase B: 0.1% (*v*/*v*) formic acid and acetonitrile-water solution (84% acetonitrile)). The chromatographic column was equilibrated with a 95% A solution. The sample was injected into the sample column (2.1 × 100 mm, 5 µm C18, Thermo) and separated on an analytical column (2.1 × 100 mm, 3 µm C18, Thermo). The flow rate was 0.25 μL/min. The linear gradient from phase A to B was 0–40%, 40–100%, and a hold at 100% at 0–50 min, 50–58 min, and 58–60 min, respectively. MS1 spectra were collected in the range of 300–1800 *m/z* for 60 ms. The 20 most intense precursors with a charge state of 2–5 were selected for fragmentation, and MS2 spectra were gained within the scope of 50–2000 *m/z* for 100 ms. Precursor ions were eliminated by reselection for 15 s.

### 4.7. iTRAQ Data Analysis

The original mass spectrometry data in the RAW files were analyzed by using Mascot (version 2.2, Matrix Science Inc, Boston, MA, USA), and the Proteome Discoverer engine (Thermo, version 1.4) was used for quantitative protein identification. The iTRAQ data were compared against the uniprot_Aspergillaceae_313392_20150818 FASTA database (31, 3392 items, updated in August 2015). The filtration parameter for the result was peptide FDR ≤ 0.01.

The biological and functional properties of the identified proteins were found by mapped protein sequences using GO terms (http://geneontology.org/). A homology search was first performed for the identified sequences with a localized NCBI BLASTP program against the NCBI database. The e-value threshold was set to less than 1e-5, and the best hit for each query sequence was taken for GO term matching. The GO term matching was performed with the Blast2GO v4.5 pipeline. The Clusters of Orthologous Groups of Proteins system (COG, http://www.ncbi.nlm.nih.gov/COG/) was used for the functional annotation of genes from new genomes to inquire into the genome’s evolution. We used a hypergeometric test to perform GO and pathway enrichment analysis to find candidate biomarkers.

iTRAQ ratios were selected for further statistical analysis if they matched the criteria of (a) being detected in all biological replicates and (b) having *p* < 0.05 using Student’s *t*-test, with a mean expression of >1.20 or <0.83 times that of the relevant control.

## Figures and Tables

**Table 1:** Ratio of *Monascus purpureus* proteins upregulated (>1.2-fold) or downregulated (<0.83-fold) identified by iTRAQ as a result of LF-MF (1.6 mT) treatment during the first two days of fermentation. *p* < 0.05.

Accession	Protein Name	Ratio
**Proteins and genes involved in citrinin biosynthesis**		
Aspzol_71435	*CtnI* (acyl-CoA synthetase)	0.629
Q4wyl4	*CtnH* (short chain dehydrogenase)	0.791
Monrupul_468568	*CtnR1* (WD repeat protein)	0.774
Evm_model_C5_699	Biotin synthetase	0.404
**Proteins and genes involved in pigment and monacolin biosynthesis**		
Pench1_76083	Polyketide enoylreductase (ER)	1.219
A0a0a2jfe4	Ketoreductase	1.208
A0a0a2lbj2penit	Esterase	1.229
A0a0a2lbj2	Esterase	1.331
Q5bcz6	*MokH* (Zn(II)_2_Cys_6_ transcription factor)	1.267
Aspwe1_68221	Fatty acid elongase	1.358
Mycgr3_71228	P450 monooxygenase	2.031
Mycgr3_98821	*mokC* (P450 monooxygenase)	1.235

## Supplementary Materials

The following is available online at http://www.mdpi.com/2072-6651/10/11/440/s1, Table S1: Differentially expressed proteins.
